# The Economic Burden of Type 2 Diabetes Mellitus in Pakistan: A Cost of Illness Study

**DOI:** 10.3390/healthcare12181826

**Published:** 2024-09-12

**Authors:** Muhammad Subhan Arshad, Faleh Alqahtani, Muhammad Fawad Rasool

**Affiliations:** 1 Department of Pharmacy Practice, Faculty of Pharmacy, Bahauddin Zakariya University, Multan 60800, Pakistan; m.subhan1995@gmail.com; 2 Department of Pharmacy, Southern Punjab Institute of Health Sciences, Multan 60000, Pakistan; 3 Department of Pharmacology and Toxicology, College of Pharmacy, King Saud University, Riyadh 11451, Saudi Arabia; afaleh@ksu.edu.sa

**Keywords:** burden of disease, diabetes, direct cost, indirect cost, Pakistan

## Abstract

Background: Type 2 Diabetes Mellitus (T2DM) is a highly prevalent disease with a chronic nature and poses a significant health burden worldwide, with no exception in Pakistan. Hence, this study aimed to explore the financial burden of T2DM in Pakistan through cost of illness analysis. Methods: A prevalence-based, cross-sectional study was conducted using a structured data collection tool from the patient’s perspective. Through structured interviews by trained data collectors, the data regarding direct medical costs, direct non-medical costs, and indirect costs were collected and further verified through prescriptions and bills. After testing the normality of data, mean and median with interquartile range were used to present cost data, while non-parametric tests, i.e., the Mann–Whitney U test and the Kruskal–Wallis test, were used to assess factors associated with costs, as cost data were not normally distributed. Results: The study included 522 participants, with a majority being female (54%) and aged between 41 and 60 years (64%). The mean annual total cost per patient was USD 235.1 (median = USD 162.8), comprising direct medical costs, 93.2% (mean = USD 219.2; median = USD 150.0), direct non-medical costs, 5.3% (mean = USD 12.4; median = USD 7.1), and indirect costs, 1.5% (mean = USD 3.5; median = USD 1.9). Costs were significantly higher for patients with advanced age, high literacy, higher household incomes, duration of diabetes, more than one complication, and using combination therapy. Conclusions: The economic burden of T2DM in Pakistan is substantial, with medication costs being the largest component. Effective management strategies and policy interventions are crucial to mitigate this burden and improve the economic and health outcomes for diabetic patients.

## 1. Introduction

Diabetes mellitus (DM) is a chronic non-communicable disease that affects millions of people and imposes a significant economic burden on individuals, healthcare systems, and national economies [1]. The burden of DM extends beyond its high prevalence rate, due to its chronic nature and associated multiple complications that act as a major contributor to the higher economic costs of DM management therapy [2]. In different types of DM, defects in insulin secretion, action, or both lead to persistent hyperglycemia. Type 2 Diabetes Mellitus (T2DM) is the most prevalent, and starts with insulin resistance [3]. Unhealthy eating habits, inactive living styles, urbanization, and aging populations are major contributing factors to the alarming increase in the prevalence of T2DM [4,5].

The International Diabetes Federation (IDF) reported that around 537 million people were living with DM worldwide in 2021, and among those 414 million people are living in middle-income countries. Pakistan is a lower-middle-income country (LMIC), where 33 million people are living with DM, with the highest comparative prevalence of 30.8% in 2021 [6]. According to IDF reports, USD 966 billion was spent on DM-related healthcare worldwide in 2021, which is expected to hike up to USD 1045 billion in 2045. Pakistan’s estimated DM-related healthcare expenditure was USD 2.6 billion in 2021 which is expected to be USD 4.4 billion in 2045 [6]. This high prevalence rate and economic burden of DM in LMIC countries like Pakistan pose a significant threat to the health and socioeconomic well-being of its population, and Pakistan is already grappling with limited healthcare resources and economic problems.

Among different methodologies that are used to estimate the economic burden of disease, cost of illness is the most common, and encompasses the direct medical cost (e.g., consultation, investigation, and medication costs) and direct non-medical cost (e.g., travel and food costs) as well as indirect cost (e.g., loss of productivity due to absenteeism and disability) [7]. In developed countries, retrospectively collected population healthcare data are commonly used to estimate the cost of disease, whereas a countrywide network of insurance companies and third-party payers properly document that healthcare utilization data alongside government healthcare departments [8,9]. Due to a lack of documentation of healthcare utilization, this methodology is not practicable in developing countries. To estimate the cost of illness in such countries, prospective surveys are conducted to collect data related to various healthcare cost factors, as carried out in various previously published studies [10,11,12,13,14].

The rapidly growing prevalence of T2DM in Pakistan is creating a significant financial impact on individuals and the healthcare system. To optimize public health and control the economic burden, comprehensive research is required to understand the economic burden of T2DM and to plan healthcare interventions. Despite existing studies, the comprehensive data on the economic burden of T2DM in Pakistan remains limited as these studies were limited to outpatient clinics [11,12] or a single city [10]. These studies were conducted in outpatient clinics, so there is a possibility that they did not approach those patients who do not regularly seek healthcare professionals’ advice or adhere to guidelines for regular check-ups. Irregular healthcare-seeking behavior and limited access to care are common among Pakistani diabetic patients, as evidenced by a recent study [15]. Hence, to address these gaps, the current study aimed to conduct a comprehensive cost-of-illness analysis to estimate the economic burden of T2DM among the diabetic patients of Pakistan, employing a community-based approach.

## 2. Materials and Methods

### 2.1. Study Design, Setting, and Population

A prevalence-based, cross-sectional cost-of-illness study was conducted to investigate the economic burden of T2DM in Punjab, Pakistan, from the patient’s perspective. According to IDF, approximately 33 million adults live with DM in Pakistan [6]. Based on this reported DM population in Pakistan, a sample size of 385 was calculated by using a Raosoft online sample size calculator with a 5% margin of error and 95% confidence level [16]. This was further inflated up to 40%, which led to the final sample size of 539, to increase the power for statistical analysis and compensate for any potential loss.

The study included diabetic patients aged 18 years and older who had been diagnosed with DM for at least one year or had been utilizing any form of antidiabetic treatment (i.e., oral medication, insulin, or both) for a minimum period of one year. All participants provided written informed consent before inclusion in the study.

Exclusion criteria were applied to patients with Gestational Diabetes Mellitus (GDM) and Type 1 Diabetes Mellitus (T1DM). Additionally, individuals diagnosed with diabetes or who had begun antidiabetic treatment less than one year before the study were excluded. Furthermore, patients who were unable to participate in structured interviews due to cognitive impairment, communication barriers, or other reasons, as well as those who failed to provide complete or verifiable data on healthcare utilization or costs, were excluded from the study.

### 2.2. Data Collection Tool and Procedure

A structured data collection tool was designed in the English language to collect the data from the targeted population, based on previously published studies with similar methodological approaches and objectives [10,11,12,17]. This structured tool comprised of questions related to socio-demographic and disease-related characteristics of patients, along with cost information. In the socio-demographic section, participants were asked about their age, gender, marital status, education level, employment status, and personal/household monthly income. The next section comprised questions to acquire data regarding the duration of diabetes, frequency of doctor visits, glycemic target monitoring practices (self-monitoring and laboratory tests), mode of treatment (i.e., diet plan/exercise, oral medications, insulin, or a combination of oral medications and insulin), presence of complications (i.e., hypertension, dyslipidemia, depression, heart disease, retinopathy, neuropathy, nephropathy), and medications used for diabetes/its complications. In the last section, to acquire cost information, participants were asked about their expenses related to consultation, laboratory tests, medications, and self-monitoring, to calculate the direct medical costs. Participants were also asked about food and travel expenses during doctor’s clinic visits to account for direct non-medical costs. In addition to that, the presence of an accompanying person along with time spent during travel, waiting, and consultancy were also inquired about to calculate the indirect cost. Prior administration to the main survey content validity of the data collection tool was assured by the experts, and a pilot study (*n* = 30) was conducted to ensure the face validity as well as for the training of data collectors. The hospitalization cost was not inquired into in the current study as the study focused on the community-based approach, where patients are not frequently admitted to the hospital, and diabetic patients with severe co-morbid conditions are usually admitted to the hospital, which could significantly alter the healthcare cost.

A convenience sampling method was utilized due to unapproachability to all statistical populations of the current study. Photocopies of prescriptions/pharmacy purchase bills and laboratory investigation reports/bills were also acquired to verify and calculate the reported cost information by the study participants. The disease-related characteristics were also confirmed by reviewing the prescriptions. The data collectors collected data through face-to-face interviews of study participants from 20 April 2024 to 10 July 2024 to achieve a desired sample size of 539. Before the start of the interview, each participant was told about the study in detail as written on the informed consent form approved by the ethics committee, and this was then signed by the participants. In the case of an illiterate participant, data collectors signed it on their behalf after seeking their permission. Participation in the study was voluntary and an interview lasted 15–20 min on average.

### 2.3. Cost Calculation

The total cost of T2DM was calculated from the primary cost information gathered via the data collection tool, comprised of direct and indirect costs as major categories. Direct cost was further sub-categorized into direct medical and direct non-medical. The direct cost was calculated using a bottom-up approach, in which direct medical cost comprised consultation cost, laboratory investigation cost, medication cost, and self-monitoring of blood glucose (SMBG) cost. In addition to that, the direct non-medical cost comprised transportation and food cost on the way to the clinic.

The micro-costing approach was utilized to find out the maximum details for calculating the direct medical cost. The annual consultation cost was calculated by multiplying the reported cost per recent visit with the number of visits reported during the last year. Annual laboratory investigation cost was calculated by multiplying the cost per test by the frequency of tests during the last year. Annual medication and SMBG costs were calculated by extrapolating the reported per-month cost in the last month to the yearly cost.

The annual direct non-medical costs were calculated by multiplying the reported expenditures on food and travel during visits to the doctor’s clinic (per recent visit) by the number of visits reported during the last year.

The indirect cost was calculated for both the patient and the accompanying person through productive time lost by both during clinic visits for consultations using the human capital approach. The time spent during traveling, waiting, and consultation during a recent visit to the clinic was recorded based on information provided by study participants, and annual productive time lost was calculated by multiplying productive time lost in that visit by the number of visits in the last year. Indirect cost was calculated by multiplying the productive time lost in hours by the value of each working hour. The value of the working day was calculated by dividing the reported monthly income by 26 working days per month, and then this daily value was divided by eight to obtain the value of each working hour. The minimum wage rate of PKR 1230.77 per day (153.85 per hour) in Pakistan was utilized to calculate productivity lost for housewives and other unemployed study participants [18].

The total cost was calculated by adding up all costs, which were calculated in Pakistani Rupees (PKR) and then converted to US Dollars (USD) using the average exchange rate for 2023 (USD 1 = PKR 280) [19].

### 2.4. Statistical Analysis

The individual data of each study participant were first entered into Microsoft Excel and then transferred to Statistical Package for Social Sciences (SPSS) version 27.0 (IBM, Armonk, NY, USA). Frequency and percentages were used to present descriptive statistical results for categorical variables. A normality test of cost data was performed using a histogram, Q-Q plot, and the Shapiro–Wilk test. Cost data were not normally distributed; hence, mean and median with interquartile range (IQR) were used to present numerical cost data. Non-parametric tests (i.e., Mann–Whitney U test and the Kruskal–Wallis test) were performed to assess the association between different groups, as cost data were not normally distributed. An alpha value of ≤0.05 was considered to be statistically significant.

## 3. Results

**Figure 1:**
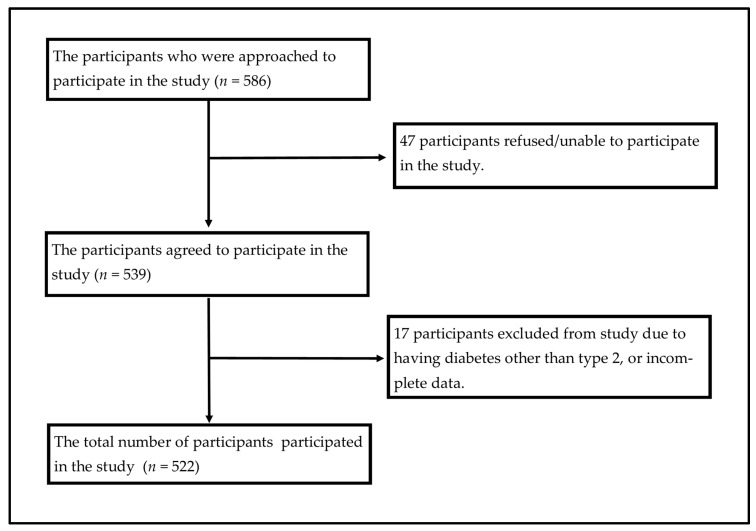
The flowchart of study participants’ recruitment.

A total of 539 DM patients were interviewed to collect the required data, among which 17 were excluded as they did not provide the required cost information or they had GDM or T1DM confirmed diagnoses written on prescriptions by their physicians (Figure 1). The study included 522 participants, with a slightly higher representation of females (54.0%) compared to males (46.0%). The majority of participants were between the ages of 41 and 60 years (64.0%), followed by those older than 60 years (18.4%) and those 18–40 years (17.6%). Most participants were married (93.5%), with a small percentage being single (3.4%) or divorced/widowed (3.1%).

Regarding education, the distribution was varied, with 26.8% having undergraduate-level education or above, 25.7% with intermediate education, and 22.2% with primary education. A notable portion of the participants were unemployed (53.3%), while 20.7% held office jobs and 11.1% worked in business. The monthly household incomes showed that 40.2% of participants earned between PKR 50,001 and 100,000, 31.8% earned more than PKR 100,000, and 28.0% earned less than PKR 50,000.

The duration of diabetes among participants was predominantly less than or equal to 5 years (62.1%), and 37.9% had diabetes for more than 5 years. Regarding complications, 37.9% of participants had more than one complication, 29.9% had one complication, and 32.2% had no complications. The mode of treatment was mostly oral medication (51.7%), followed by combination therapy (27.2%), insulin (15.3%), and diet/exercise (5.7%) (Table 1).

The median annual consultation cost per person was USD 8.9, with the 25th and 75th percentiles at USD 5.4 and USD 21.4, respectively. Investigation costs were higher, with a median of USD 42.9, ranging from USD 14.3 to USD 64.3. Medications represented the highest direct medical cost, with a median of USD 128.6, ranging from USD 64.3 to USD 214.3, and the annual cost for SMBG was USD 42.9. In terms of direct non-medical costs, travel and food costs were both at a median of USD 5.4. The total direct cost amounted to a median of USD 158.6. Productivity loss for patients was minimal at USD 0.8, while the loss for attendants was slightly higher at USD 1.7, leading to a total indirect cost of USD 1.9. The overall total cost per person was USD 162.8, with a range between USD 107.5 and USD 302.9. Details can be seen in Table 2.

**Figure 2:**
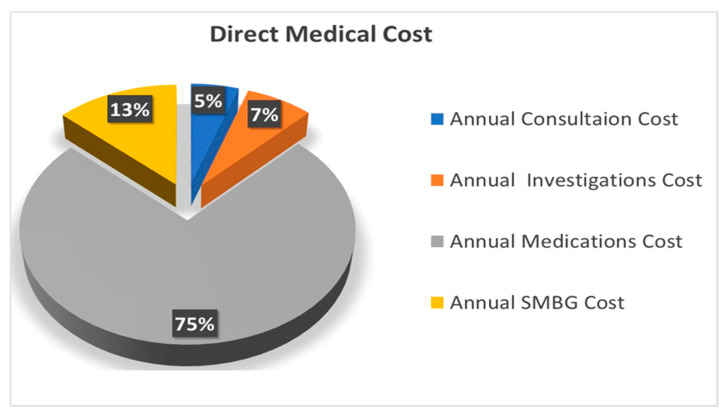
Percentage breakdown of direct medical cost of T2DM among study participants.

**Figure 3:**
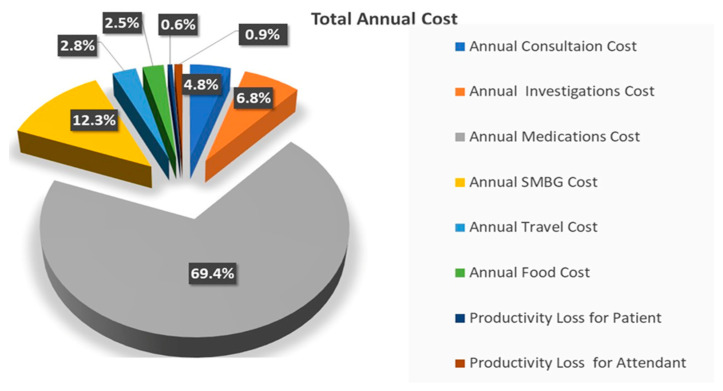
Percentage breakdown of total cost of T2DM among study participants.

The distributions of the direct medical costs and the overall total annual costs for T2DM are presented in Figure 2 and Figure 3, respectively. Medications represented the largest financial burden, constituting 74% of the total direct medical cost and 69.4% of the overall total annual cost. SMBG accounted for 13% of the direct medical cost and 12.3% of the total annual cost. Investigations comprised 7% of the direct medical cost and 6.8% of the overall total annual cost, while consultations accounted for 5% and 4.8%, respectively. The smallest contributions to overall total annual cost came from productivity losses, which accounted for 0.6% and 0.9% for patients and attendants, respectively.

The costs associated with T2DM varied significantly based on different demographic and socio-economic factors: Females had a higher median total cost (USD 186.2) compared to males (USD 151.4). Participants older than 60 years had the highest total cost (USD 229.7), followed by those aged 41–60 years (USD 153.2) and 18–40 years (USD 158.9). Divorced/widowed participants faced significantly higher costs (USD 617.4) than married (USD 158.4) or single individuals (USD 102.7). Participants with graduate-level education and above incurred the highest costs (USD 289.8), and illiterate participants had the lowest (USD 128.8). Businesspeople and those with office jobs had higher costs (USD 225.9 and USD 184.6, respectively) compared to laborers and unemployed individuals. Higher-income groups (>PKR 100,000) faced the highest total costs (USD 270.9). Those with diabetes for more than 5 years had higher costs (USD 186.2) compared to those with diabetes for ≤5 years (USD 153.2). Participants with more than one complication had the highest costs (USD 200.9). Combination therapy incurred the highest costs (USD 273.2), while diet/exercise had the lowest (USD 43.7). The details can be seen in Table 3.

## 4. Discussion

The chronic nature of DM and its progressively increasing prevalence has made it a major contributor to the global economic burden, which can be reduced by the proper management of the main contributing factors to its high management cost. Cost of illness analysis provides a basic understanding of various components of the cost that contribute to the economic burden of disease, which can be utilized to plan strategies to reduce the financial impact of disease. Hence, the current study explored the economic burden of management of T2DM in Pakistan utilizing cost of illness methodology. The total mean annual cost per person was USD 235.1, with mean annual direct costs amounting to USD 231.6 and mean annual indirect costs, mainly due to productivity loss, amounting to USD 3.5. These results also highlighted the significant financial impact on individuals due to direct medical costs, amounting to about USD 219.2 with the majority contribution by medication costs (USD 163.2).

The mean total annual cost for each T2DM patient explored in the current study is comparatively lower than those in previously published studies from Pakistan, which reported USD 740 in 2022 [12] and USD 555 in 2016 [11]. A possible explanation for this variation could be the difference in the estimated cost components, as the study with the highest reported cost included the hospitalization cost, which was excluded from our study. The variation from the study with second-highest reported cost could be due to the methodology adopted by them to calculate annual cost. They calculated total annual costs by the multiplication of recorded total monthly costs (i.e., the sum of all costs components) by the number of months in a year, whereas in the current study, we used different approaches as per the need of the cost components. The difference in reported costs could also be due to a probable variation in consultancy fees originating from the methodological approach for data collection, as these previously published studies were conducted at private outpatient clinics. Our study, however, utilized a community-based approach to capture a diverse population, taking doctors’ consultations from both private and public sector healthcare facilities. The annual mean total cost reported by the study conducted in the outpatient clinics of different healthcare settings from Karachi, Pakistan, was USD 197 in 2006, which is lower than the mean total annual cost calculated in the current study. A possible reason for the difference in the reported cost could be inflation occurring during recent years.

Studies with similar perspectives and comparable methodologies from different countries in the region reported a total mean cost of T2DM ranging from the lowest, USD 142 in Iran [20], to the highest, USD 314 and USD 409 in Bangladesh [14,17], while USD 246 was found in Vietnam [21]. The median total costs of diabetes management alone and with comorbidities reported by the community-based study from India were USD 73 and USD 133, respectively [13]. The cost reported by the study from Vietnam is consistent with our study. This variation from other studies could be due to the difference in reporting year and exchange rates of local currencies with USD, as well as due to differences in medication prices and costs for healthcare services across these countries. The medication cost, as a component of direct medical cost, is the highest contributor to the total cost of diabetes without hospitalization costs across all the studies in the region, with no exception for the current study. Advanced age, higher monthly household income, longer duration of diabetes, presence of complications, and a combination mode of treatments were commonly reported as factors significantly associated with the higher cost, which is consistent with our study.

The high costs associated with T2DM in Pakistan were largely driven by expenses related to medication, aligning with findings from other regional studies. Beyond the direct financial burden on individuals, the economic impact of T2DM extends to the broader healthcare system and workforce productivity. The increasing prevalence of T2DM strains the already-limited healthcare resources, leading to higher healthcare expenditure and potentially compromising the quality of care for other conditions. Additionally, the indirect costs associated with productivity losses, including absenteeism and reduced work capacity due to diabetes-related complications, significantly contribute to the overall economic burden on society. These factors underscore the urgent need for comprehensive public health strategies that not only to address the medical management of T2DM but also its broader societal implications, including workforce productivity and healthcare system sustainability.

The study has several limitations that should be acknowledged. First, the data were collected through a community-based approach using a convenience sampling method, which was chosen due to the inaccessibility of certain populations for randomization. This approach may have introduced selection bias, potentially affecting the generalizability of the findings to the entire country. Second, the reliance on self-reported data for cost estimates introduces the risk of recall bias, particularly in cases where participants may have had difficulty in accurately remembering past expenses. Third, the study does not account for potential variability in costs across different healthcare providers, which could influence the accuracy of the overall cost estimates. This variability may be significant, as costs can differ widely between public and private healthcare settings, as well as across different regions. Fourth, the study lacks a sensitivity analysis, which would have allowed for an assessment of the robustness of the cost estimates under different assumptions, particularly regarding indirect costs that are highly variable. Future research should aim to include a more diverse and randomized sample, consider the variability of costs across different healthcare settings, and incorporate sensitivity analyses to better understand the uncertainty and potential bias in cost estimates. These enhancements would help to improve the accuracy and generalizability of the findings.

## 5. Conclusions

The present study provides a detailed analysis of the economic burden of T2DM in Pakistan, with the total mean annual cost per patient estimated at USD 235.1 (median = USD 162.8), comprising 93.2% direct medical costs, 5.3% direct non-medical costs, and 1.5% indirect costs. Patients with advanced age, higher literacy, higher household incomes, longer duration of diabetes, multiple complications, and combination therapy had significantly higher costs.

In addition to the findings, this study suggests several recommendations and areas for future research. First, there is a critical need for policy interventions aimed at subsidizing the cost of medications, which constitute the largest portion of the economic burden. Enhancing public health initiatives focused on early diagnosis and effective management of diabetes, particularly in underserved regions, could also help to reduce long-term costs. Future studies should explore the impact of implementing community-based diabetes education programs on reducing direct and indirect costs. Moreover, longitudinal studies are recommended to track the long-term economic impact of T2DM. Research into the variability of the expenses across different healthcare settings, as well as the potential for integrating cost-saving opportunities in diabetes management, would also be valuable in informing policy and healthcare practices.

## Figures and Tables

**Table 1:** Demographic, socio-economic, and disease-related characteristics of the study participants (*n* = 522).

		Frequency	Percentage
Gender	Male	240	46.0%
	Female	282	54.0%
Age	18–40 Years	92	17.6%
	41–60 Years	334	64.0%
	>60 Years	96	18.4%
Marital Status	Single	18	3.4%
	Married	488	93.5%
	Divorced/Widowed	16	3.1%
Education Level	Illiterate	70	13.4%
	Primary	116	22.2%
	Secondary	62	11.9%
	Intermediate	134	25.7%
	Graduate and Above	140	26.8%
Employment Status	Office Job	108	20.7%
	Business	58	11.1%
	Laborer	50	9.6%
	Unemployed	278	53.3%
	Other	28	5.4%
Monthly Household Income	≤PKR 50,000 (USD 178)	146	28.0%
	PKR 50,001 to PKR 100,000 (USD 179 to USD 357)	210	40.2%
	PKR 100,0000 (USD 357)	166	31.8%
Duration of Diabetes	≤5 Years	324	62.1%
	>5 Years	198	37.9%
Number of Complications	No Complications	168	32.2%
	1 Complication	156	29.9%
	>1 Complications	198	37.9%
Mode of Treatment	Diet Plan/Exercise	30	5.7%
	Oral Medication	270	51.7%
	Insulin	80	15.3%
	Combination	142	27.2%

Note: PKR = Pakistani Rupee, USD = US dollar.

**Table 2:** Annual cost of Type 2 Diabetes Mellitus per person in USD.

Cost Components		Mean	Median	25th Percentile	75th Percentile
Direct Medical Cost	Annual Consultation Cost	11.3	8.9	5.4	21.4
	Annual Investigations Cost	15.9	42.9	14.3	64.3
	Annual Medications Cost	163.2	128.6	64.3	214.3
	Annual SMBG Cost	28.8	42.9	21.4	64.3
Total Direct Medical Cost		219.2	150.0	107.1	278.6
Direct Non-Medical Cost	Annual Travel Cost	6.6	5.4	2.5	10.7
	Annual Food Cost	5.8	5.4	1.8	10.7
Total Direct Non-Medical Cost		12.4	7.1	3.6	16.1
Total Direct Cost		231.6	158.6	107.1	300.0
Indirect Cost	Productivity Loss for Patient	1.3	0.8	0.4	1.4
	Productivity Loss for Attendant	2.2	1.7	1.0	3.4
Total Indirect Cost		3.5	1.9	1.0	3.7
Total Cost		235.1	162.8	107.5	302.9

Note: SMBG = self-monitoring of blood glucose, USD = US Dollar.

**Table 3:** The association of annual direct, indirect, and total costs (USD) of Type 2 Diabetes Mellitus with characteristics of the study participants.

		Direct Cost			Indirect Cost			Total Cost		
		Median	IQR	*p*-Value	Median	IQR	*p*-Value	Median	IQR	*p*-Value
Gender	Male	150.0	107.1, 263.8	**0.013**	1.6	0.9, 3.9	0.119	151.4	107.5, 268.6	**0.016**
	Female	182.1	107.1, 375		2.1	1.1, 3.5		186.2	107.5, 380.3	
Age	18–40 Years	156.3	100, 269.6	**0.033**	1.7	0.9, 2.9	**0.013**	158.9	101.8, 272.2	**0.034**
	41–60 Years	150.0	107.1, 285		1.9	1.1, 3.9		153.2	107.5, 286.9	
	>60 Years	228.6	126.8, 364.3		2.1	1.1, 3.8		229.7	127.6, 369.9	
Marital Status	Single	101.8	64.3, 230.4	**<0.001**	0.9	0.8, 1.6	**0.033**	102.7	65.6, 232	**<0.001**
	Married	154.6	107.1, 284.4		1.9	1.1, 3.8		158.4	107.5, 286.1	
	Divorced/Widowed	614.3	489.3, 675		2.7	2.2, 3.4		617.4	492, 677.6	
Education Level	Illiterate	128.6	64.3, 159.6	**<0.001**	1.3	0.5, 1.9	**<0.001**	128.8	65.6, 162.6	**<0.001**
	Primary	146.4	100, 261.8		2.0	0.8, 3.1		150.6	104.3, 264.2	
	Secondary	121.4	78.6, 219.6		1.7	1.1, 3.6		126.3	80.3, 225.9	
	Intermediate	150.0	107.1, 285		1.5	1, 3.4		151.5	107.5, 286.9	
	Graduate	285.7	165, 439.3		3.1	1.7, 5.3		289.8	169, 443	
Employment Status	Office Job	179.5	125, 300	**0.002**	2.6	1.5, 4.3	**<0.001**	184.6	126.2, 302.9	**0.001**
	Business	219.6	146.4, 371.4		3.9	1.4, 5.8		225.9	150.3, 374	
	Laborer	128.6	100, 167.9		0.9	0.6, 2		129.5	102.1, 169	
	Unemployed	150.0	100, 316.8		1.8	0.8, 3.2		150.5	104.3, 324.7	
	Other	150.0	90.7, 283.6		1.6	0.9, 1.8		152.4	92.4, 285.9	
Monthly Household Income	≤PKR 50,000 (USD 178)	107.1	64.3, 138.2	**<0.001**	1.2	0.6, 1.8	**<0.001**	108.9	65.9, 139	**<0.001**
	PKR 50,001 to PKR 100,000 (USD 179 to USD 357)	162.5	107.1, 263.2		2.1	1.1, 3.2		164.6	107.5, 264.3	
	>PKR 100,0000 (USD 357)	267.9	150, 464.3		3.5	1.6, 6.3		270.9	153.2, 476.1	
Duration of Diabetes	≤5 Years	150.0	102.5, 262.5	**0.012**	1.9	0.9, 3.3	0.609	153.2	105.1, 265.5	**0.012**
	>5 Years	182.1	118.6, 385.7		1.8	1.1, 3.8		186.2	122, 389	
No. of Complications	No Complications	134.6	93.2, 254.5	**<0.001**	1.9	0.7, 3.2	0.093	135.8	96.6, 257.5	**<0.001**
	1 Complication	160.7	107.1, 261.8		1.8	1, 3.3		163.5	108.3, 264.2	
	>1 Complications	189.3	118.6, 375		2.1	1.3, 4.4		200.9	126.2, 380.3	
Mode of Treatment	Diet Plan/Exercise	42.9	21.4, 137.1	**<0.001**	1.3	0.5, 1.9	**0.024**	43.7	21.9, 138.3	**<0.001**
	Diabetes Tablets	147.9	101.8, 255.4		2.0	1.3, 3.8		150.3	104.5, 257.3	
	Insulin	148.2	110.7, 283		1.1	0.4, 3		150.7	111.1, 285.8	
	Combination	269.6	162.5, 514.3		2.1	1.1, 3.9		273.2	164.6, 522.9	

Note: Statistically significant results are highlighted in bold font; IQR = interquartile range.

## Data Availability

The data that support the findings of this study are available from the corresponding author upon reasonable request.
